# National Commissions on High Quality Health Systems: activities, challenges, and future directions

**DOI:** 10.1016/S2214-109X(18)30528-X

**Published:** 2019-02

**Authors:** National Commissioners on High Quality Health Systems, Harvard T.H. Chan

**Affiliations:** Department of Global Health and Population; School of Public Health, Boston, MA 02115, USA

The Lancet Global Commission on High Quality Health Systems in the Sustainable Development Goal era (HQSS Commission) demonstrated that health care without quality is ineffective, wasteful, and hampers progress towards improved health outcomes.^1^,^2^ Although national governments recognise the need to invest in high-quality health systems, many existing national strategies are weak, fragmented, or poorly implemented. In order to foster more sustainable quality initiatives,^3^,^4^ it is important to examine the experiences that countries have faced on the path towards high quality health systems. Guided by the global HQSS Commission, the National Commissions on High Quality Health Systems (National Commissions) were established in 2017 in nine countries (Argentina, Ethiopia, Malawi, Mexico, Nepal, Philippines, Senegal, South Africa, and Tanzania) to organise national work on quality, contribute to the overall findings of the HQSS Commission, and build capacity for quality measurement and analysis within countries (appendix). We identified common themes in the experiences reported by the various National Commissions in relation to activities, key accomplishments, challenges, and future directions. The findings highlighted key elements in delivering high-quality care, which will be useful for national-level policy making and cross-country learning.

The National Commissions have worked in three key areas: local evidence generation and translation to policies and practices, creating a platform for discussing quality, and capacity building towards delivering quality care (appendix). First, National Commissions have worked on collating and producing local evidence on quality, providing baseline quality of care information, and examining how such evidence can be best translated to policies and practices. Prior to the National Commissions, some countries did not have an institution dedicated to quality of care. In Mexico for example, the National Commission did the first nationwide online survey designed using the global HQSS Commission framework to describe foundations for quality of care in their country and identify barriers to quality evaluation and improvement. In Tanzania, the Commission’s work promoted inclusion of quality of care metrics in the government’s star rating system, wherein facilities were scored on the basis of set health system performance metrics. In addition to using the global HQSS Commission framework as a guide in selecting national quality indicators, other government agencies percieved the Ethiopia Commission to be well-positioned for producing reputable and unbiased evidence. Second, National Commissions served as springboards for institutionalising quality initiatives in the political agenda. In South Africa, the Commission found that enabling legislation is not sufficient to guarantee implementation and must be accompanied by specific efforts to enable implementation of the legislation to create changes in quality of care. The Ethiopia Commission mandated regular multisectoral meetings, in which the latest evidence was discussed to inform quality improvement initiatives. The Philippines Commission meetings were focused on updating existing national policy on quality. Thus, some National Commissions have worked to underscore quality as a priority in the bigger political agenda. Through promoting a common understanding of quality concepts, metrics, and design strategies, these groups can enable minimum quality standards to be implemented in their countries. Lastly, the HQSS Commission facilitated capacity-building through information and skills sharing, and integration of isolated quality efforts. Prior to the Commission, quality initiatives were mostly done without coordination with other stakeholders within and across countries. The Argentina Commission is examining South Africa’s Ideal Clinic Realisation and Maintenance programme,^5^ which is designed to improve the quality of primary health care services, to offer practical ideas that could also be implemented in Argentina. The Nepal Commission is also intending to collaborate with other countries on their quality initiatives. Thus, the community of National Commissions has allowed for integration across previously isolated quality efforts.

Nevertheless, the National Commissions have met several challenges. First, in many countries, the absence of a single institution responsible for initiatives of health system quality reduced accountability for quality of care, as well as transparency, as experienced by the Philippines and Mexico Commissions. The various quality of care initiatives in different institutions also led to each organisation having a separate agenda on quality that was not necessarily linked to national policies. As such, the Tanzania National Commission considered shifting their Commission to a government steering committee to ensure better coordination, accountability, transparency and sustainability of quality initiatives. Second, the National Commissions, particularly in Nepal, struggled to unify the diverse actors essential to sustaining and expanding quality initiatives. Inevitably, challenges remain in bridging between academics and policy makers, as well as other actors. Policies on quality of care were mostly decided by high-level organisations, particularly the government, with limited input from other actors. These government-led initiatives were reportedly different from the National Commissions’ recommendations, with the National Commissions more concerned about long-term rather than short-term quality of care outcomes. In Malawi and Ethiopia, the National Commissions found that their public health programmes were focused on disease-specific quality initiatives that aimed to improve clinical practices, rather than systemwide interventions such as training of health workers. For the Philippines National Commission, existing initiatives were focused more on improving process standards and hospital infrastructures according to the International Organization for Standardization, rather than a more patient-centred quality assurance approach to care. The Argentina and Mexico Commissions also found that the involvement of local authorities has been weak compared with that of the national authorities in their countries. Thus, quality initiatives remain fragmented, leading to difficulties in ensuring a common vision for high quality health systems.

To move the quality of care discussions forward, National Commissions from Argentina, Tanzania, and the Philippines, among others, intend to institutionalise their Commissions as part of the government to ensure inclusion and prioritisation of quality of care, particularly when updating existing policies and quality assessment tools. Other National Commissions, including Senegal’s, will further expand quality of care evidence. The National Commissions intend to further collaborate with other Commissions and other stakeholders for better multisectoral and cross-country learning. Future efforts will aim to position quality of health systems as a priority in the political agenda, alongside universal health coverage.

The National Commissions have facilitated quality of care discussions within countries, and increased the priority of health system quality in the political agenda. Specifically, the National Commissions have contributed to evidence generation and translation, institutionalisation of quality initiatives and other programmes, information and skills transfer among different health system actors, and capacity building. However, challenges remain for the National Commissions, particularly in generating political will and engagement with strengthening quality of care, harmonising various fragmented quality health systems to support initiatives, and sustaining the momentum of quality of care improvements in their countries. Future work of the National Commissions will focus on further building the evidence base on quality of care, prioritising quality of care in their existing public health programmes, and developing existing collaborations with other relevant health system actors and countries.

## Supplementary Material

Supplementary appendix 1
