# Supplementary material for: National Commissions on High Quality Health Systems: activities, challenges, and future directions
Source: Lancet Glob Health. Author manuscript; Available in PMC 2019 Feb 15. (PMC6377060; doi:10.1016/S2214-109X(18)30528-X)

THE LANCET
Global Health

Supplementary appendix

This appendix formed part of the original submission and has been peer reviewed. We post it as supplied by the authors.

Supplement to: National Commissioners on High Quality Health Systems. National Commissions on High Quality Health Systems: activities, challenges, and future directions. *Lancet Glob Health* 2019; **7:** e179–80.

Erlyn K. Macarayan, PhD [a,b]

Argentina National Commission:

Ezequiel García-Elorrio, MD PhD [c]

Viviana Elizabeth Rodriguez, PhD [d]

Maria Eugenia Teijeiro, MD [e]

Ethiopian National Commission:

Bereket Yakob, PhD [f]

Kaleab Baye, PhD [g]

Kebede Deribe, PhD [h]

Mexican National Commission:

Svetlana V Doubova, MD [i]

Sebastián García-Saisó, MD [j]

Ricardo Pérez-Cuevas, MD [k]

Nepal National Commission:

Amit Aryal, MPH [l]

Philippine National Commission:

Beverly Lorraine Ho, MD MPH [m]

Senegal National Commission:

Youssoupha Ndiaye, MD PhD [n]

Ndella Konate, MD MPH [o]

South African National Commission:

Prof Laetitia Rispel, PhD [p]

Thulani Masilela, MA [q]

Tanzanian National Commission:

Talhiya Yahya, MD [r]

Mohamed A. Mohamed, PhD [s]

Eliudi Eliakimu, MD [t]

Global Commission:

Anna Gage, MSc [a]

Affiliations:

a Department of Global Health and Population, Harvard T.H. Chan School of Public Health, 665 Huntington Avenue, Boston, MA 02115, USA

b Ariadne Labs, Brigham and Women's Hospital & Harvard T.H. Chan School of Public Health, 3rd Floor East Wing, 401 Park Drive, Boston MA 02215 Boston, MA, USA

c Director. Departamento de Calidad de Atención Médica y Seguridad del Paciente/ Director Health Care Quality and Patient Safety, Instituto de Efectividad Clínica y Sanitaria/ Institute for Clinical Effectiveness and Health Policy, Buenos Aires, Argentina

d Coordinadora Calidad de Atención Médica y Seguridad del Paciente/ Coordinator. Health Care Quality and Patient Safety, Instituto de Efectividad Clínica y Sanitaria/ Institute for Clinical Effectiveness and Health Policy, Buenos Aires, Argentina

e Jefe de Calidad y Acreditación, FLENI Escobar. Chief Quality officer, FLENI Escobar Buenos Aires Argentina

f Evidence to Policy Specialist, Fenot Project, Harvard TH Chan School of Public Health, Department of Global Health and Population, Addis Ababa, Ethiopia

g Associate Professor, Director, Center for Food Science and Nutrition, College of Natural and Computational Sciences, Addis Ababa, Ethiopia

h Research Fellow, Department of Global Health and Infection, Brighton and Sussex Medical School, University of Sussex, Brighton, United Kingdom

i Epidemiology and Health Services Research Unit, Mexican Institute of Social Security, Mexico City, Mexico

j Ministry of Health, Mexico City, Mexico

k Health System Research Center, National Institute of Public Health, México City, Mexico

l Advisor to Member of Parliament Gagan K Thapa, Federal Parliament of Nepal, Kathmandu, Nepal

m Department of Health, Manila, Philippines

n Director for Planning, Research, and Statistics, Ministry of Health and Social Action, Dakar, Senegal

o Quality Program Coordinator, Ministry of Health and Social Action, Dakar, Senegal

p DST/NRF Research Chair, School of Public Health, University of Witwatersrand, Johannesburg, South Africa

q Department of Planning, Monitoring and Evaluation, Presidency, Pretoria, South Africa

r Head, Quality Management Unit Coordinator – Star Rating of Health Facilities, Health Services Inspectorate and Quality Assurance Section, Health Quality Assurance Division, Ministry of Health, Community Development, Gender, Elderly and Children, 6 Samora Machel Avenue PO Box 9083 Dar Es Salaam, Tanzania

s Health Quality Assurance Division, Ministry of Health, Community Development, Gender, Elderly and Children, PO Box 743, Dodoma, Tanzania

t Assistant Director, Health Sciences Inspectorate and Quality Assurance Section, Health Quality Assurance Division, Ministry of Health, Community Development, Gender, Elderly and Children, PO Box 743, Dodoma, Tanzania

List of National Commissioners

South Africa
Prof. Olive Shisana
Prof. Laetitia Rispel
Prof. Ames Dhai
Prof. Lilian Dudley
Dr. Rene English
Dr. Gerhard P Grobler
Thulani Masilela
Dr. Rajesh Patel
Prof. Adrian Puren
Russell Rensburg
Jacqui Stewart
Prof. Stuart Whittaker
Dr. Gustaaf G Wolvaardt

Ethiopia
Dr. Ephrem Tekle
Prof Tsinuel Girma
Dr. Bereket Yakob
Dr. Girmaye Dinsa
Dr. Amare Deribew
Dr. Kebede Deribe
Dr. Kalaeb Baye
Sibhatu Biadgilign
Atkure Defar
Dr. Della Berhanu
Theodros Getachew
Tariku Dejene
Dr. Seifu Hagos
Fasil Tessema
Dr. Kassahun Asheber
Dr. Araya Abrha Medhanyie
Haftom Gebrehiwot
Dr. Lisanu Tadesse
Girum Taye
Dr. Tadesse Alemu
Ermias Dessie

Tanzania
Dr. Mohamed A. Mohamed
Dr. Ntuli Kapologwe
Dr. Subi Leon
Dr. Kyungu
Thomas Teuscher
Norzin Grigoleit- Dagyab
Honarati Masanja
Gayle Martin
Mrs. Miwa Ito
Theopista John
Dr. Asia Hussein
Hassan Mshinda
Julius Massaga
Prof. Muhsin Aboud
Dr. Samwel Ogillo
Dr. Eliudi Eliakimu
Dr. Talhiya Yahya

Senegal
Dr. Marie Khemesse Ngom
Dr. Youssoupha Ndiaye
Dr. Ndella Konate
Prof. Yerim Mbagnick Diop
Ms. Coumba Aby Sarr
Dr. Abdoulaye Bousso
Dr. Ahmadou Bouya Ndao
Dr. Papa Abdoulaye Seck
Prof. Halimatou Diop-Ndiaye
Mr. Ibrahima Ndour
Dr. Evariste Lodi

Philippines
Dr. Lilibeth C. David
Dr. Oscar F. Picazo
Dr. Agnes D Mejia
Dr. Leonila F Dans
Dr. John Q Wong
Mike Santos
Dr. Francisco Soria
Dr. Beverly Lorraine C. Ho
Dr. Louella Patricia Carpio

Mexico
Dr. Sebastián García Saisó
Dr. Svetlana V. Doubova
Dr. Ricardo Pérez Cuevas
Dr. Odet Sarabia González
Mtra. Paulina Pacheco Estrello
Dr. Adolfo Martínez Valle
Dr. Brisa Itzel Amparán Limas
Dr. Claudia Infante Castañeda
Dr. Carmen Santa Maria Guasch
Dr. Laura del Pilar Torres Arreola
Dr. Pedro Saturno Hernandez

Malawi
Dr. Andrew Likaka
Prof. Address Malata
Prof. Wilson Mandala
Prof. Ellen Chirwa
Mrs. Harriet Chanza
Dr. Isabel Kazanga
Ms. Hsin-yi Lee
Dr. Damson Kathyola
Dr. Atnaf Getachew Asfaw
Vandana Stapleton
Mr. Isaac Dambula
Ms. Kari Edvardsdal Hansen
Prof. William Stones
Dr. Bejoy Nambiar
Mr. Saulos Mhlanga
Dr. Owen Chikhwaza
Dr. Queen Dube
Eric Tsetekani
Dr. Ruth Hildebrandt
Martha Chidzuma Mwangonde
Dr. Martias Joshua
Dr. Phylos Bonongwe

Nepal
Dr. Ramesh Kant Adhikari
Dr. Dipendra Raman Sing
Mr. Amit Aryal
Dr. Suresh Mehata
Dr. Kishori Mahat
Dr. Ghanashyam Gautam
Mr. Sudip Pokhrel
Dr. Ruma Rajbhandari
Ms. Franziska Fuerst
Mr. Ian Chadwell
Dr. Sushil Baral
Ms. Aradhana Thapa
Mr. Sudip Pokhrel
Dr. Runa Jha
Dr. Sameer Dixit
Dr. Ashish KC
Mr. Sagar Dahal
Ms. Ranju Sharma

Argentina
Dr. Hugo Arce
Dr. Nora Dackiewicz
Dr. Ricardo Durlach
Dr. Ezequiel García Elorrio
Dr. Ana Fajreldines
Dr. Ricardo Armando Otero
Ariel Alejandro Palacios
Dr. Viviana E Rodriguez
Dr. Eduardo Schnitzler
Dr. María Eugenia Teijeiro
Dr. Fabián Vitolo

Acknowledgments

We acknowledge The Lancet Global Health Commission on High Quality Health Systems Secretariat, particularly Margaret Kruk for her substantial input to the work and for all the support provided. Each National Commission would like to acknowledge the following: Argentina National Commission (under the name of Argentinian Group for Quality of Care): Hugo Arce, Nora Dackiewicz, Ricardo Durlach, Ana Fajreldines, Ezequiel Garcia Elorrio, Ricardo Otero, Ariel Palacios, Marcelo Pellizzari, Viviana Rodríguez, Eduardo Schnitzler, Eugenia Teijeiro, Fabián Vitolo; Ethiopian National Commission: Ephrem Tekle Lamango, who actively worked as a global commissioner, as well as facilitating the work of the Ethiopia National Commission; Mexico National Commission: Paulina Pacheco-Estrello and Odet Sarabia-González from the Directorate for Quality of Healthcare and Education, Ministry of Health, Mexico and Claudia Infante-Castañeda from the Institute of Social Research, National Autonomous University of Mexico, Mexico; Senegal National Commission: Abdoulaye Ly and Marie Khemesse Ngom Ndiaye, Health General Director, Ministry of Health and Social Action, Dakar, Senegal. South Africa National Commission: Ames Dhai, Lilian Dudley, Rene English, Gerhard Grobler, Rajesh Patel, Adrian Puren, Russell Rensburg, Jacqui Stewart, Stuart Whittaker, Olive Shisana, Gustaaf Wolvaardt. KD is funded by a Wellcome Trust International Intermediate Fellowship (grant number 201900).

National Commissions on High Quality Health Systems: current progress and next steps

Key activities of the National Commissions	Generate evidence:<br>• Used the global HQSS Commission framework on high quality health systems to: guide the development of surveys and other data collection instruments in the country (Mexico), gather baseline data on quality of care (Mexico), strengthen current quality of care activities at the ministry of health (Mexico), and guide data analysis to measure quality (Ethiopia)<br>• Provided evidence on quality, as well as policy briefs, that can be used for policy formulations and in developing national programs on quality (Ethiopia, Senegal)<br>• Developed locally relevant metrics on quality of care (Ethiopia, Tanzania). The Tanzanian government aims to have 80% of primary health facilities above a 3-star rating by mid-2018 and efforts are now underway to ensure inclusion of quality of care metrics when assessing health facilities useful in pinpointing best and worst performers.<br>Bring together dispersed actors:<br>• Initiated discussions on quality health systems at the national level (Argentina, Ethiopia, Senegal)<br>• Served as a springboard for dialogues among local experts and other health system actors to build capacity for synthesis and use of evidence for policymaking, program development, and other health system strengthening initiatives (Ethiopia, Mexico, Senegal, and South Africa)<br>• Served as a platform for cross-country learning (Argentina, Mexico)<br>• Ensured political commitment for quality of care through establishing quality-related priority programs (Senegal)<br>Build capacity:<br>• Built capacity for skill- and information-sharing for quality of care within the country (Ethiopia)<br>• Cemented the capacity of the Ministry of Health Quality Assurance Division in analysis of the data collected and information sharing across stakeholders (Tanzania)<br>• Amplified the work of other relevant quality stakeholders (South Africa through the Office of Health Standards Compliance)
Challenges	Prior to the Commission, there were:<br>• No universal definitions on quality of care (Senegal)<br>• No clear framework on quality and if there were, the frameworks were limited or were disease-specific rather than having a system-wide approach on quality of care (Philippines, South Africa)<br>• No clear existing benchmarks and metrics on quality of care in the country (Ethiopia, Philippines). In the Philippines, quality of care data was coming from providers rather than observations of care or patient exit interviews; hence, was an incomplete and one-sided picture of quality.<br>• No baseline information on disparities and gaps in quality health care services (Ethiopia)<br>• No minimum standards on quality of care (Ethiopia)<br>• Isolated quality initiatives and efforts (Senegal)<br>With the National Commissions formed, their ongoing challenges include:<br>• Some local authorities and public sectors were difficult to engage on discussions on quality (Mexico)<br>• Lack of a unified vision around quality health care (Nepal)<br>• Bureaucracies in government and different political priorities (Argentina, Philippines, Mexico)<br>• Lack of institutions or organizations that are accountable on ensuring quality of care across the country (Philippines, South Africa)<br>• Policy translation - difficulties in influencing the government on major design of national policies on quality or in implementing quality initiatives (Argentina, South Africa)
Future directions and intentions	Research<br>• Gather additional data on quality and analyze existing available data (Ethiopia, Senegal). The Senegal Commission will work on projects assessing quality of primary health care with the Primary Health Care Performance Initiative (PHCPI), as well as on quality of maternal and child care with the World Bank to be launched in 2019. Other planned projects in Senegal include assessing users' perceptions on quality of care, and improving the Standard Operating Procedures for data quality audits, among others.<br>• Initiate new research to monitor the progress toward UHC or to address knowledge gaps (South Africa)<br>Learn<br>• Continuously create dialogues on quality bridging different stakeholders (Ethiopia, Mexico, Nepal, Senegal)<br>• Adapt the global framework and tools on high quality health systems and apply to local contexts (Ethiopia, Mexico, Philippines, Tanzania, Senegal)<br>• Collaborate with other countries and/or institutions on quality of care initiatives (Nepal, Mexico, Tanzania)<br>Influence policy<br>• Evolve the National Quality Policy and Strategy to acknowledge new priorities and systemic challenges (Mexico)<br>• Integrate quality as a priority in the health sector plans and agenda and in designing future quality of care programs/interventions (Ethiopia, Mexico, Philippines, Senegal, South Africa)

	• Raise the quality of health care as a political agenda through increased citizen engagement (Nepal, México)<br>• Develop national priorities on quality (Argentina)<br>• Develop a National Quality Improvement Plan with clear milestones and timeframes (South Africa)<br>Sustain<br>• Institutionalize a National Commission on quality (Argentina, Ethiopia, Tanzania)<br>• Source funding to continue the Commission's work (Malawi, Senegal)

National Commissions on High Quality Health Systems in Nine Countries

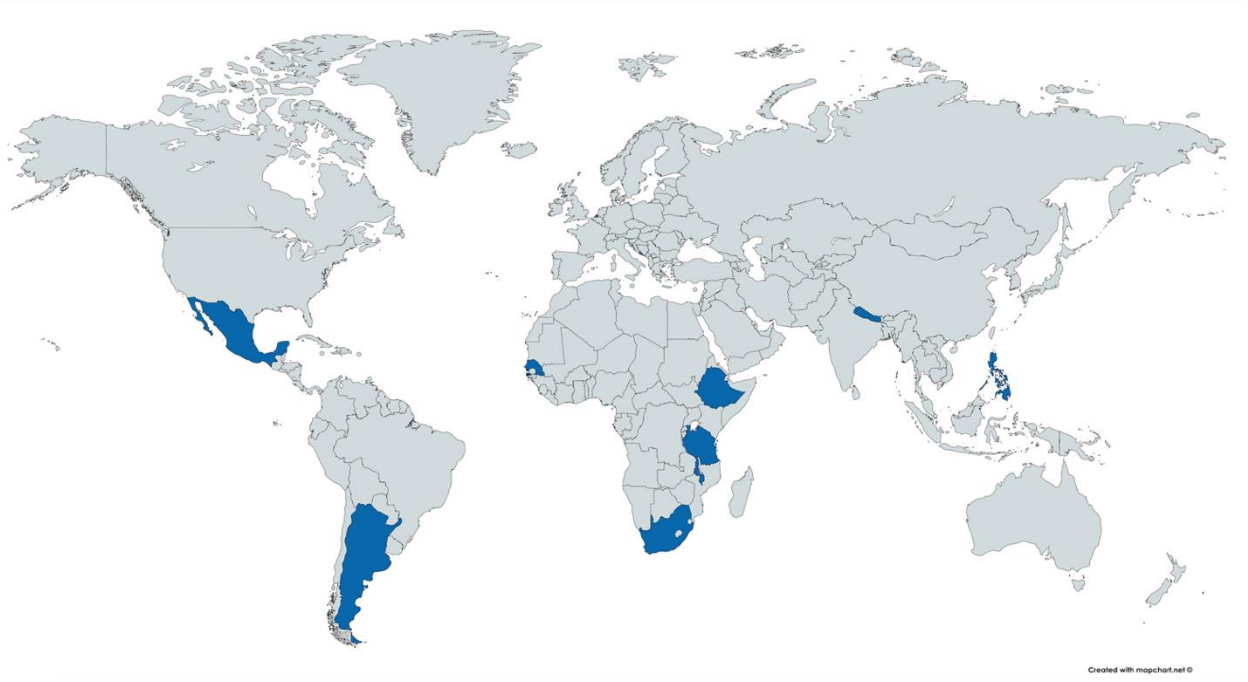

Supplement: Supplementary appendix 1 [file NIHMS81708-supplement-Supplementary_appendix_1.pdf]
